# Left hepatic trisectionectomy for hilar cholangiocarcinoma presenting with an aberrant biliary duct of segment 5: a case report

**DOI:** 10.1186/1752-1947-4-250

**Published:** 2010-08-06

**Authors:** Nobuhisa Akamatsu, Yasuhiko Sugawara, Masahiko Komagome, Takashi Ishida, Nobuhiro Shin, Narihiro Cho, Fumiaki Ozawa, Daijo Hashimoto

**Affiliations:** 1 Department of Hepato-biliary-pancreatic surgery, Saitama Medical Center, Saitama Medical University, 1981 Tsujido-cho, Kamoda, Kawagoe, Saitama 350-8550, Japan; 2 Artificial Organ and Transplantation Division, Department of Surgery, Graduate School of Medicine, University of Tokyo, 7-3-1 Hongo, Bunkyo-ku, Tokyo 113-8655, Japan

## Abstract

**Introduction:**

Management of the biliary ducts during liver resection is one of the most important challenges for hepatobiliary surgeons. Here, we report the case of a left hepatic trisectionectomy for hilar cholangiocarcinoma with a rare aberrant biliary duct of segment 5, which, to the best of our knowledge, has never been reported in previous literature.

**Case presentation:**

A 56-year-old Asian female initially presented with intrahepatic bile duct dilatation in the left lateral sector, left paramedian sector, and right paramedian sector. Simultaneous cholangiography from a percutaneous transhepatic biliary drainage tube in biliary duct of segment 8 and endoscopic nasobiliary drainage tube in biliary duct of segment 3 revealed drainage of the right lateral sectoral branch into the common hepatic duct and the aberrant drainage of segment 5 into the right lateral sectoral branch. The left hepatic duct, right paramedian sectoral duct, and the confluence of the right lateral sectoral duct were narrowed. Left hepatic trisectionectomy was successfully performed with careful dissection and division of the aberrant biliary duct of segment 5.

**Conclusion:**

For safe liver resection, it is important to perform a detailed anatomic evaluation of the intrahepatic ducts, both preoperatively and intraoperatively.

## Introduction

Advances in surgical procedures for liver resections and partial liver transplantation have led to the need for a better, more detailed understanding of biliary anatomy and potential variations to perform a safe operation. Management of the biliary ducts during liver resection is one of the most important challenges for hepatobiliary surgeons. The biliary anatomy is variable: 24% to 57% of individuals have variant biliary patterns [1-6]. Most variant cases involve right-lobe drainage that typically arises from an anomalous insertion of the right lateral sectoral duct (draining Couinaud's segments 6 and 7) into the left hepatic duct, common hepatic duct, or common bile duct, among others [1-5].

We recently experienced a case of a Klatskin tumor with rare biliary anatomy that, to our knowledge, has not been reported previously, and we present the case herein.

## Case presentation

**Figure 1:**
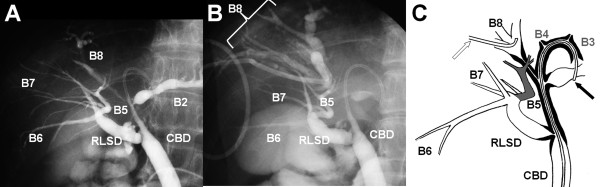
**Biliary images of this case**. **(a) **Preoperative cholangiography from a endoscopic nasobiliary drainage (ENBD) tube inserted into B3. **(b) **Simultaneous cholangiography via the ENBD and percutaneous transhepatic biliary drainage (PTBD) tubes. The PTBD was inserted into B8. **(c) **A schematic of the biliary tree of this case. The ENBD tube was inserted into B3 (black arrow). The PTBD tube was inserted into B8 (white arrow). The lesions are marked in black. Aberrant B5 is marked in gray. B3 and B4 were not opacified because of severe stenosis.

A 56-year-old Asian woman was admitted to our hospital for bile-duct dilatation in the left lateral sector, left paramedian sector, and the right paramedian sector. First, an endoscopic nasobiliary drainage tube was inserted into the left hepatic duct, and then a percutaneous transhepatic biliary drainage tube was inserted into the right paramedian sectoral biliary duct from the tributary of segment 8. Simultaneous cholangiography from the percutaneous transhepatic biliary drainage and endoscopic nasobiliary drainage tubes revealed drainage of the right lateral sectoral branch into the common hepatic duct and the aberrant drainage of segment 5 into the right lateral sectoral branch. Ductal narrowing was observed in the left hepatic duct, the right paramedian sectoral duct, and the confluence of the right lateral sectoral duct and was assumed to be due to hilar cholangiocarcinoma (Figure 1). No other anomaly was observed.

In view of the biliary anatomy, resection of segments 1 to 4 and 8 (that is, a left trisectionectomy preserving segment 5) was considered, but the portal branch of segment 5 originated from the root of the right paramedian branch, which precluded preservation of the portal pedicle of segment 5 for a curative operation. Consequently, a conventional left hepatic trisectionectomy was planned for curative surgery in this case, and preoperative portal vein embolization of the left portal vein and the right paramedian sectoral branch was performed to increase the parenchymal volume of the right lateral sector.

**Figure 2:**
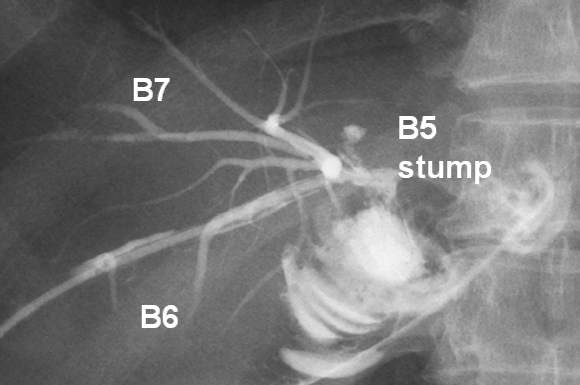
**Postoperative cholangiography**. Postoperative cholangiography from the retrograde transhepatic biliary drainage tube (RTBD), which was inserted into B6.

Finally, a left hepatic trisectionectomy was successfully performed. During dissection of the liver parenchyma, the aberrant biliary duct of segment 5 (B5) was isolated and divided. Biliary reconstruction was performed by using a hepatico-jejunostomy with a retrograde transhepatic biliary drainage tube. The pathologic investigation of the specimen confirmed hilar cholangiocarcinoma, with negative surgical margins. The postoperative cholangiography from the retrograde transhepatic biliary drainage tube is shown in Figure 2. The patient was discharged on postoperative day 42 without biliary complications and is alive without recurrence 4 years after the operation.

## Discussion

The aberrant B5 from the anomalous right lateral sectoral branch, which joined with the common hepatic duct, was the novel finding in this case. Intrahepatic biliary duct variations are usually classified as one of five types, according to the insertion point of the right lateral sectoral duct (Table 1) [1-5].

**Table 1:** Conventional and common variations of biliary anatomy according to the insertion of the right lateral sectoral duct

Type	Anatomical comments
I	Conventional bifurcation type; right lateral sectoral duct joins the right paramedian duct to form the right hepatic duct, and then finally, the right and left hepatic ducts join to form the common hepatic duct
II	Trifurcation type; right lateral sectoral duct joins the confluence of the right paramedian sectoral duct and the left hepatic duct to form a trifurcation
III	Right lateral sectoral duct joins separately to the left hepatic duct
IV	Right lateral sectoral duct joins separately to the common hepatic duct
V	Right lateral sectoral duct joins separately to the cystic duct

Many reports have addressed these variations, but few have reported variations of the segmental biliary ducts. To our knowledge, this is the first report of an aberrant B5 originating from the right lateral sectoral duct. Puente *et al. *[2] retrospectively reviewed 4264 intraoperative cholangiograms and reported that accessory B6 joined the common bile duct or cystic duct in 76 (1.9%) cases. Choi *et al. *[5] observed 16 (5%) cases with an accessory B6 that joined the right hepatic duct or common hepatic duct among 300 consecutive living partial-liver donors. Mortele and colleagues [6] reported anatomic variants of the biliary tree based on magnetic resonance cholangiograms and reported an accessory B8 joining the right lateral sectoral duct and an accessory B2 that joined the right paramedian sector in conjunction with an aberrant bile duct. They emphasized the importance of recognizing these anomalies to avoid postoperative bile leakage. Huang *et al. *[1] retrospectively reviewed 958 endoscopic retrograde cholangiograms and discussed the aberrant insertion of B4, in which B4 occasionally joined the right hepatic duct or B2. They emphasized that surgeons should be aware of these ductal variants in left lateral sectorectomy and left lobectomy. B2 and B3 [5] and B5 and B8 [7], separately joining to the common bile duct, were reported in a living donor liver transplantation.

In terms of the preoperative recognition of bile-duct anatomy, multi-detector computed tomography scanning after drip infusion cholangiography and magnetic resonance cholangiography [8] are equivalent for detecting secondary branching with satisfactory accuracy, but the accurate detection of tertiary branching, even with recent advances of these modalities, is difficult [5]. For biliary evaluation in association with hilar cholangiocarcinoma, despite recent reports emphasizing the efficacy of multi-detector computed tomography [9], direct cholangiography remains the gold standard for the preoperative evaluation of longitudinal ductal spread of the lesion [10]. Unfortunately, only an eight-row computed tomography and direct cholangiography were available for this case, and we used the direct cholangiography as the reference standard with satisfactory results.

## Conclusion

Surgeons might encounter any imaginable bile-duct variation and so detailed preoperative and intraoperative anatomic evaluation of the intrahepatic ducts is important for safe bile drainage after surgical resection.

## Abbreviations

Bn: biliary duct of Couinaud's segment n.

## Competing interests

The authors declare that they have no competing interests.

## Authors' contributions

AN and SY interpreted the patient images regarding the biliary anatomy. AN performed the operation and was a major contributor to writing the manuscript. All authors read and approved the final manuscript.

## Consent

Written informed consent was obtained from the patient for publication of this case report and accompanying images. A copy of the written consent is available for review by the Editor-in-Chief of this journal.
